# Signs of Dehydration after Hip Fracture Surgery: An Observational Descriptive Study

**DOI:** 10.3390/medicina56070361

**Published:** 2020-07-18

**Authors:** Louise Ekman, Peter Johnson, Robert G. Hahn

**Affiliations:** 1 Department of Geriatrics, Dalens Hospital, 121 31 Enskededalen, Sweden; louise.m.ekman@gmail.com; 2 Department of Geriatrics, Nacka Clinic, 131 37 Nacka, Sweden; peter.johnson@capio.se; 3 Department of Research, Södertälje Hospital, 152 86 Södertälje, Sweden; 4 Karolinska Institutet at Danderyds Hospital (KIDS), 182 57 Danderyd, Sweden

**Keywords:** urine color, dehydration, frail elderly patient, urine osmolality

## Abstract

*Background and Objectives:* Dehydration might be an issue after hip fracture surgery, but the optimal tools to identify the dehydrated condition have not been determined. The aim of the present study was to compare the characteristics of elderly postoperative patients who were classified as dehydrated according to the methods used in the clinic. *Materials and Methods:* Thirty-eight patients aged between 65 and 97 (mean, 82) years were studied after being admitted to a geriatric department for rehabilitation after hip fracture surgery. Each patient underwent blood analyses, urine sampling, and clinical examinations. *Results:* Patients ingested a mean of 1,008 mL (standard deviation, 309 mL) of fluid during their first day at the clinic. Serum osmolality increased significantly with the plasma concentrations of sodium, creatinine, and urea. Seven patients had high serum osmolality (≥300 mosmol/kg) that correlated with the presence of tongue furrows (*p* < 0.04), poor skin turgor (*p* < 0.03), and pronounced albuminuria (*p* < 0.03). Eight patients had concentrated urine (urine-specific gravity ≥ 1.025) that correlated with a low intake of liquid and with a decrease in body weight during the past month of −3.0 kg (25–75 th percentiles, −5.1 to −0.9) versus +0.2 (−1.9 to +2.7) kg (*p* < 0.04). *Conclusions:* Renal fluid conservation of water, either in the form of hyperosmolality or concentrated urine, was found in 40% of the patients after hip fracture surgery. Hyperosmolality might not indicate a more severe fluid deficit than is indicated by concentrated urine but suggests an impaired ability to concentrate the urine.

## 1. Introduction

Hip fracture is associated with a 1-year mortality of between 10% and 30% [1,2]. The surgical repair is a serious event in the life of the senior citizen, who may need postoperative rehabilitation in a geriatric clinic. The rehabilitation might be prolonged by dehydration, which provokes changes in cardiovascular, thermoregulatory, metabolic, and central nervous functions; impairment of physical performance is often reported even with dehydration of only 2% of the body mass [3]. Cognitive abilities and mood states are positively influenced by water consumption, while the opposite effects are particularly relevant for those with poor fluid regulation, such as in the elderly [4]. 

Dehydration is a challenging diagnosis in the geriatric population, as symptoms are usually vague and may only consist in a low systolic blood pressure [5]. Clinical tests have been difficult to evaluate, as no biochemical “gold standard” exists that tells us whether a patient is dehydrated or not. Blood and urine analyses have been used, but interpretation is hampered by the multifactorial settings that are typical for postoperative geriatric patients.

A raised plasma osmolality is the most widely used standard reference test for poor intake of water, but its usefulness has been uncritically extrapolated from healthy athletes to all age groups [5]. An alternative test is based on urine sampling. Increased concentrations of metabolic waste substances are found in the urine when the kidneys conserve water, which is an early response to dehydration. With losses of body water, the urine color gradually darkens, the osmolality and the creatinine concentration increase, and the specific gravity of the urine becomes higher. Concentrated urine is a valid index of exercise-induced dehydration up to 69 years of age [6], while its usefulness to detect chronic dehydration in an aged population is still uncertain.

In the present study, we used multiple approaches to study dehydration in patients under in-hospital rehabilitation after hip fracture surgery. The aim was to compare clinical signs and observations, as well as blood and urine analyses, were compared to suggest how the different markers of dehydration should be interpreted, as well as to allow a statement to be made about the prevalence of dehydration. The hypothesis was that hyperosmolality indicates a more severe form of dehydration than is indicated by concentrated urine, which has a high sensitivity to detect renal water conservation [6].

## 2. Materials and Methods

### 2.1. Patients and Ethical Statement

Between May and October 2018, patients admitted to the Department of Geriatrics at Dalen Hospital in Stockholm, Sweden, for rehabilitation after hip fracture surgery participated in a one-arm uncontrolled, unblinded observational study of dehydration. The study comprised background data taken from the hospital medical records, clinical assessment of dehydration, blood and urine sampling, and recording of perceived thirst. The Ethics Committee of Stockholm had approved the protocol (2018/462-32 on 6 March 2018, officer in charge Agneta Nordenskjöld). Informed consent was obtained from subjects with no cognitive impairment. For those with dementia, consultation was done with the relatives who were informed in the same way after the patient had accepted inclusion.

### 2.2. Data Collection and Analyses

Upon admission of the patient, the medical record was reviewed for the day of surgery, number of drugs, main and secondary diagnoses, and the last recorded body weight. The first author (LE) performed a physical examination that included non-invasive arterial pressure, heart rate, tissue turgor, thirst (visual analogue scale from 0 to 100), presence of urinary catheter, and inspection of the oral mucous membranes and the tongue for dryness and longitudinal furrows. The body weight was measured again, and to the nearest 0.1 kg, after placing the patient in a chair scale dressed in light clothing.

The intake of food and water over the first 24 h after admission was recorded. The intake of food was taken as the fraction of the served meals that was ingested, as recorded on a scale ranging from 0% to 100% by the staff working with the patient. The water consumption was noted on a “fluid list” placed by the staff beside the patient’s bed.

Blood and urine were sampled by a nurse. Patients who could not leave a urine sample went through a bladder scan to exclude urinary retention. If this was the case, the bladder was catheterized. One urine sample was immediately evaluated by LE for visual estimation of color [7], and the urine specific gravity was measured on a Clinitek Status Analyzer (Siemens, Frimley, UK) with Multistix dipsticks. The other samples were sent to the certified Unilabs at St Göran’s Hospital in Stockholm for analysis of serum osmolality and the plasma concentrations of sodium, potassium, creatinine, urea, C-reactive protein, and albumin.

Urine was further analyzed for osmolality and the concentrations of sodium, potassium, creatinine, and albumin. The Fluid Retention Index, which is a summary measure of four urinary indices of renal water conservation (color, osmolality, urine-specific gravity, creatinine), was calculated as previously described [8].

### 2.3. Statistics

The data were expressed as the mean and standard deviation (SD), except for the use of the median value and (25–75 th percentiles) when the data had a skewed distribution. No formal power analysis was made, as the study was of explorative nature. Differences in continuous parameters between sub-groups were studied by one-way analysis of variance (ANOVA), while skewed distributions were assessed using Mann–Whitney’s U test. Differences in the distribution of categorical variables were studied by contingency table analysis. Correlations between parameters were studied by simple linear regression, where r = the correlation coefficient. The regression line is curvilinear in case the relationship was logarithmic, while the graphs are always scaled in real values. *p* < 0.05 was considered significant.

## 3. Results

### 3.1. Demographics

Thirty-eight subjects, 17 males and 21 females, were studied. They were aged 82 ± 9 (range 65–97) years and had undergone surgery for acute hip fracture 5 (4–8) days earlier.

There were 21 lateral and 17 medial hip fractures, of which one was pathological. Treatment consisted in nail 14, screw 5, plate 6, and partial 7 or total hip replacement 6.

Eight patients had heart failure, 4 used diuretics, 6 had dementia, and another 3 had mild cognitive disturbance. Eight patients had an indwelling bladder catheter, which correlated with a higher number of chronic medications, 12 versus 9 (*p* < 0.04), but not to other variables.

### 3.2. General Observations

Table 1 shows the blood and urine analyses, clinical indices, and hemodynamics.

The patients ingested 1,008 ± 309 mL of liquid during the first day after admission. They ate most of the food they were served, but between-patient variability was great (median 96%, 25–75 th percentiles 67–100).

The breathing frequency increased slightly with the heart rate (r = 0.34, *p* < 0.04). The mean arterial pressure was significantly lower in the few patients with mild cognitive disturbance (69 ± 7 mmHg) and in those treated with diuretics (72 ± 8 mmHg) when compared to the other patients (86 ± 8 mmHg; both differences *p* < 0.03).

Age and gender were not statistically associated with these measurements, and no dehydration variable was statistically associated with any of the hemodynamic variables.

### 3.3. Linear Correlations

**Figure 1:**
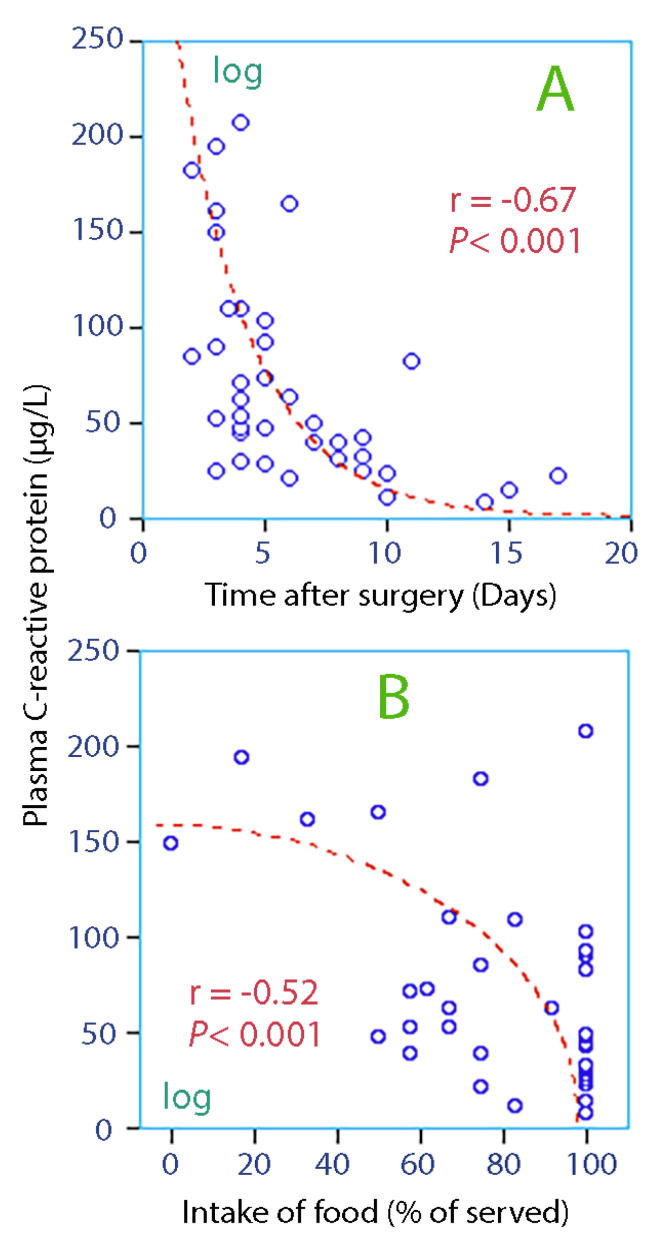
Biomarker of inflammation vs. time after surgery (**A**) and intake of food (**B**). Data could be logarithm-transformed or square root-transformed, as indicated on the appropriate axis, before being tested for correlation by linear regression analysis.

The patients had inflammatory activation, as evidenced by an increased plasma concentration of C-reactive protein (Figure 1A).

The intake of food on the first day of admission correlated significantly with the C-reactive protein concentration (Figure 1B).

**Figure 2:**
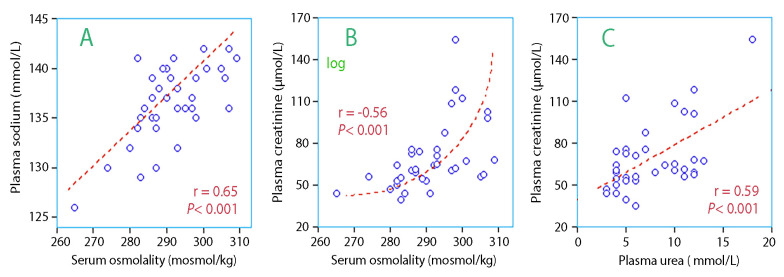
Serum osmolality vs. biomarkers of kidney function. (**A**) plasma sodium, (**B**) creatinine, and (**C**) creatinine vs. urea).

Serum osmolality increased with plasma sodium (Figure 2A) and plasma creatinine (Figure 2B). Plasma urea also increased with plasma creatinine (Figure 2C). These relationships show that hyperosmolality usually co-existed with an impairment of the kidney function. In contrast, there was no correlation between the creatinine concentration in plasma and urine, which suggests that highly concentrated urine and hyperosmolality are unlikely to be present in the same patient.

**Figure 3:**
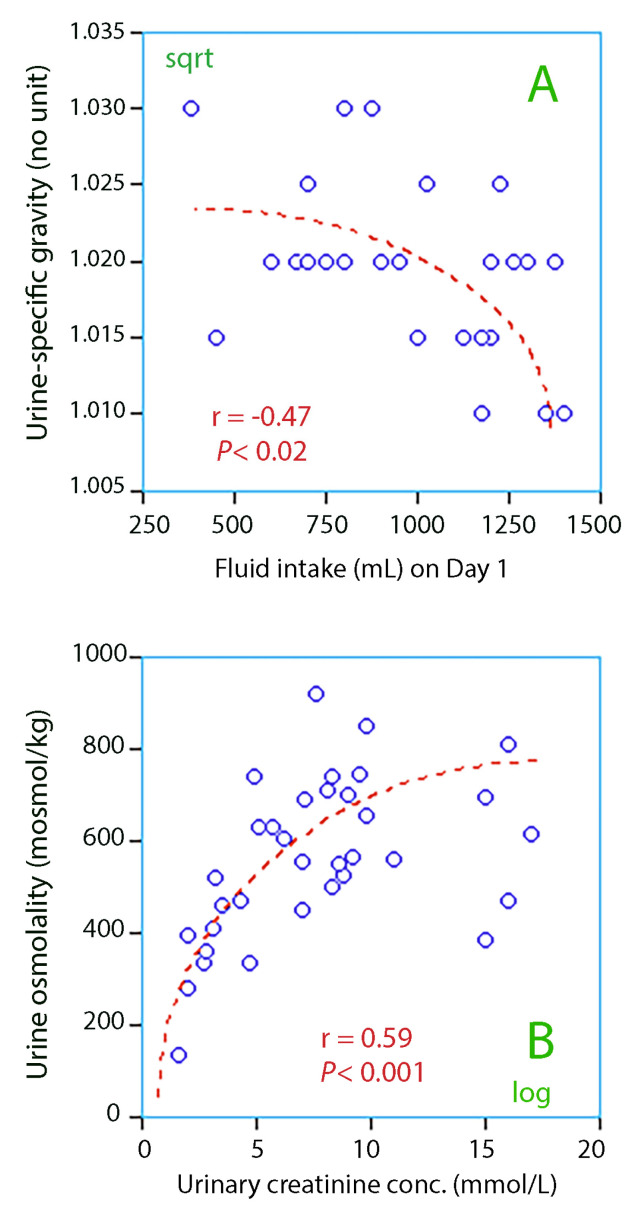
(**A**) Lower urine-specific gravity for higher fluid intake; (**B**) Correlation between urine osmolality and urinary creatinine.

Low fluid intake on the day of admission was associated with high urine-specific gravity, which was the key marker of dehydration (Figure 3A).

There were statistically significant linear relationships between the four urinary markers of dehydration, which were urine-specific weight and the urine color, osmolality, and creatinine. The logarithmic relationship between urine creatinine and urine osmolality highlights the difficulty for the elderly to manage their osmotic balance when other urinary markers of dehydration show that the kidneys strongly conserve water (Figure 3B).

### 3.4. Indices of Dehydration

Patients were dichotomized depending on whether they were considered to have hyperosmolality, ingested low amounts of fluid, or had concentrated urine.

#### 3.4.1. Hyperosmolality

Patients with hyperosmolality (defined as ≥300 mosmol/kg, *n* = 7) drank less fluid than others, median 785 versus 1044 mL, but this difference was not statistically significant. However, they had higher plasma sodium, 139.9 (3.8) versus 135.7 (2.0) mmol/L (*p* < 0.01; Figure 2A) and also had more pronounced albuminuria than the others, 9.9 (5.0–12.1) versus 2.6 (1.2–7.5) mg/mmol (*p* < 0.03).

Tongue furrows were frequent (in 6/7 patients, *p* < 0.04). Only 6 of all patients had poor skin turgor, but 3 of them had hyperosmolality (*p* < 0.032).

#### 3.4.2. Low Fluid Intake

**Figure 4:**
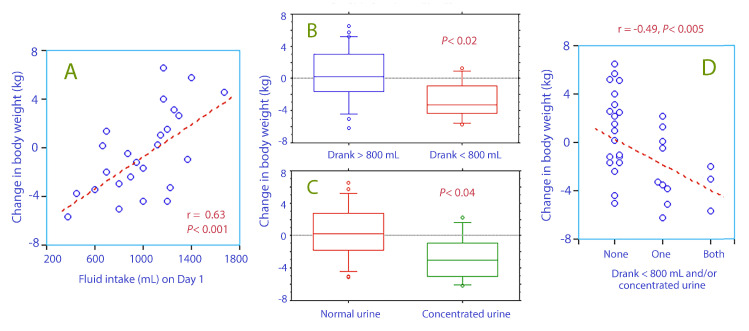
The change in body weight during the past 6 weeks (mean, 38 d) vs. (**A**) the intake of fluid on the day of admission on a linear scale (**B**) whether fluid intake was higher or lower than 800 mL on the day of admission (**C**) whether the urine was normal or concentrated (urine-specific gravity ≥1.025), and (**D**) whether none, one of both of these two variables was present in the patient.

The 9 patients who drank ≤800 mL of liquid on the day of admission had recently lost body weight, as shown by our comparison data from the admission and a previous one recorded 38 (10–150) days earlier (Figure 4A,B).

Patients with low fluid intake were less thirsty than those who drank more (visual analogue scale, median core 30 versus 41) and tended to have higher plasma sodium (mean 138.3 versus 135.9 mmol/L), but these differences were not statistically significant.

#### 3.4.3. Concentrated Urine

The 8 patients with concentrated urine (urine-specific gravity ≥1.025) had lost more body weight since the measurement made 38 days earlier (Figure 4C). They also had a lower fractional sodium excretion, 0.30 (0.22–0.44) versus 0.58 (0.31–0.88) (*p* < 0.02).

#### 3.4.4. Agreement between Methods

Hyperosmolality was present in 33% of the patients with low intake of fluid and in 38% of those with concentrated urine. Concentrated urine occurred in 38% of those with low intake of fluid (Figure 4D).

#### 3.4.5. Fluid Retention Index

In an explorative analysis, the four indices of concentrated urine were summarized as the Fluid Retention Index [6,8,9].

Nineteen patients (51%) had a score of ≥4.0, which is the cut-off for dehydration in sports medicine [6].

These patients ate less of the served food the first day, 67 (28)% versus 89 (19)% (*p* < 0.01). They also had lower plasma albumin, 24.1 (3.4) versus 26.6 (3.4) g/L (*p* < 0.04), and higher plasma sodium, 138.4 (2.6) versus 135.1 (4.1) g/L (*p* < 0.01), as well as lower fractional sodium excretion, 0.33 (0.25–0.50)% versus 0.61 (0.45–0.93)% (*p* < 0.001).

Plasma creatinine did not differ between the two groups.

Only 1 of the 8 patients with heart failure had FRI ≥4.0 (*p* < 0.031).

## 4. Discussion

### 4.1. Setting

The present results were collected in the presence of inflammatory activation due to hip fracture surgery, which apparently had a time course of approximately 2 weeks (Figure 1A). Poor appetite was the most apparent effect of the inflammation, while the sources of dehydration had to be searched elsewhere.

Several methods were available to evaluate dehydration, but the most relevant approach in a geriatric population has been difficult to identify. The classical way is to measure serum osmolality. Observing the fluid intake in the hospital can also be used, where low intakes might indicate established dehydration or dehydration under development. Concentrated urine may be used to quantify acute fluid deficits during exercise in middle-aged [6] and young [10,11] subjects, but its value in identifying chronic dehydration in the geriatric population is still unclear.

### 4.2. Hyperosmolality

Serum osmolality exceeded 300 mosmol/kg in almost 20% of our patients.

The relationships shown in Figure 2 link hyperosmolality to impaired kidney function. Those who developed hyperosmolality had apparently impaired capacity to concentrate the urine. Kidney injury is also implicated by the presence of more severe albuminuria in these patients.

The fact that elevated plasma creatinine prevented the urine from becoming highly concentrated opens the possibility that dehydration can be indicated either by hyperosmolality or concentrated urine. Therefore, these indices correlate poorly, as previously reported in another study [9].

Figure 2A suggests that two-thirds of the hyperosmolality was due to high concentrations of extracellular ions, which dehydrate the cells.

### 4.3. Low Fluid Intake

The second index of dehydration, low fluid intake, tells a slightly different story. Those who drank only small amounts of liquid were not thirsty and had lost weight during the preceding 6 weeks, suggesting a catabolic state. Plasma sodium was in the upper part of the normal range, but hyperosmolality had only developed in a few of them.

The normal daily intake of fluid in middle-aged humans is between 2.2 and 2.5 L [12]. The minimal amount is 1 mL/kg/h, which would be 1.6 L in the present cohort. Our patients drank only 1 L per 24 h on average, which is normally compensated by concentrating the urine to an osmolality of 800 mosmol/kg. Many of the patients were apparently not capable of concentrating their urine that much (Figure 3B). Those who became hyperosmotic might lack this capacity, and some may even have become hyperosmotic, although their habitual intake of water that well exceeded 800 mL per day.

### 4.4. Concentrated Urine

Concentrated urine also indicated liquid intake on the low side (Figure 3A) and recent weight loss (Figure 4C) but also a low fractional sodium excretion, which suggests that a sodium-sparing mechanism operated. Hence, the present study provides evidence that concentrated urine is due to underhydration in the elderly and possibly also to a catabolic state, while the integrity body fluid volumes were still well defended by a sufficiently effective renal function. However, as long as the water intake is low, the body´s capacity to compensate for low water intake might be exhausted at any time and promote the development of hyperosmolality, regardless of whether impaired kidney function is a consequence of underhydration or not. In the present cohort, 22% of the patients were in this risk zone.

We also performed an extended analysis of concentrated urine by applying a composite index: the Fluid Retention Index. Again, these low-water intake patients appeared to be dehydrated and even catabolic, although they had not yet developed hyperosmolality. We found no evidence of impaired kidney function being an issue.

### 4.5. Physical Examination

Physical examinations have long been used to detect dehydration in the clinic. Hyperosmolality was the only index of dehydration that could be indicated by the clinical examination of poor skin turgor and tongue furrows with any degree confidence. Vivanti et al. have found that examination of skin turgor and tongue dryness is of value [13,14], but this view is not consistent [9,15]. The correlations, if any, have never been strong.

### 4.6. Thirst

Although many signs of dehydration were found, none of them was associated with increased thirst. Patients probably received water according to their perceived need, but the self-reported thirst intensity still averaged somewhat higher than in a nursing home population (41 versus 34; [9]) and was clearly higher than in elderly patients without heart failure admitted to acute hospital care (thirst intensity, 25; [16]) or in young volunteers (thirst intensity, 11; [17]).

### 4.7. Laboratory Analyses

The laboratory methods used to assess dehydration have been developed in acute medicine and from experiments in athletes, but their usefulness in geriatric medicine is uncertain. Studies of urinary plasma biomarkers were initiated 25 years ago in sports medicine [7], and these have not been investigated until recently as a surrogate measure of the fluid balance in the general population [18]. Concentrated urine, but not hyperosmolality, is common in healthy hospital workers [8], and studies link concentrated urine to a low daily intake of water [19]. Urinalysis has also been applied to hospital care, and it apparently has relevance to clinical outcomes. For example, concentrated urine is associated with a high 30-day mortality in acute geriatric care [20].

A general opinion is that fluid intake should be sufficiently large to maintain a Fluid Retention Index of 4 or less, which corresponds to urine osmolality <600 mosmol/kg, urine-specific gravity of ≤1.020, urine color ≤4 and urine creatinine of ≤12 mmol/L. However, only a few studies have recently suggested that concentrated urine is a risk factor for poor clinical outcomes in the elderly [20,21].

### 4.8. Limitations

The limitations of the present study include its small and unselected cohort and that the groups were not randomized. The evaluation was based on a search for associations and differences between variables that could increase our understanding of dehydration in the elderly. This approach is time-consuming and may seem speculative, but other strategies are difficult to apply when a “gold standard” does not exist.

This study identified no risk groups for dehydration, but few diagnoses were tested (heart failure, dementia, mild cognitive disturbance, and use of diuretics). This result still indicates that the care was good for those who would have an increased likelihood of developing dehydration. More risk groups were screened for dehydration in a previous study of 256 patients, but only those with confusion had more concentrated urine than the other patients [20].

The urine sample was not taken after an overnight fast, which would be optimal, but it was always collected after complete fasting for several hours. We refrained from using the Fluid Retention Index as the key index for concentrated urine because correlations between the parameters are not as linear in geriatric care [20] as they are in middle-aged volunteers [8]. In particular, the ability to concentrate the urine is poorer and the muscle mass is smaller in the elderly than in the young, making urine osmolality and creatinine measurements less valid. The scores for dark urine were higher than in previous work. Urine-specific gravity seems to be the most consistently trustworthy biomarker [10], and it was used as the key measure of concentrated urine as well in the present study.

Our figures for water intake did not include metabolic water, which contributes 250–300 mL per day. The food also contains water and accounts for up to 25% of the total intake. Several observations are missing, as disclosed by sparse data in some of the graphs. The strengths of the study include the evaluations of all patients by the same physician (LE) and the involvement of only one hospital.

## 5. Conclusions

In the study population, 40% of the patients had either hyperosmolality or concentrated urine, with limited overlap, as signs of dehydration. Patients who drank little (<800 mL/day) or had concentrated urine had also lost several kilos in body weight during the past month. Urine-specific gravity and serum osmolality seem to be the most valuable biomarkers of low intake of water in the elderly postoperative patient. Our results suggest that hyperosmolality does not indicate a more severe water deficit than is indicated by concentrated urine; rather, it indicates a poorer renal capacity to handle a low fluid intake.

## Figures and Tables

**Table 1:** **Clinical assessment data.** Data showing a normal distribution is given as mean (SD). Skewed distributions are reported and the median (25–75 th percentile).

	Mean or Median	SD or 25–75 th Percentiles	Extreme Values
**Blood analyses**			
Plasma albumin (g/L)	26	4	≤25 (*n* = 18)
Plasma sodium (mmol/L)	136.5	3.8	≤130 (*n* = 4)
Plasma potassium (mmol/L)	3.9	0.5	2.8 and 5.3
Plasma creatinine (µmol/L)	68	25	>100 (*n* = 6)
Plasma C-reactive protein (µg/L)	61	21–92	>100 (*n* = 9)
Plasma urea (mmol/L)	6	5–11	>10 (*n* = 10)
Serum osmolality (mosmol/kg)	291	286–298	299 (*n* = 7) and 265 (*n* = 1)
Fractional sodium excretion (%)	0.47	0.28–0.72	<0.3 (*n* = 10)
**Urine analyses**			
Urine osmolality (mosmol/kg)	548	149	132 to 916
Urine creatinine (mmol/L)	7.8	4.2	>10 (*n* = 8)
Urine potassium (mmol/L)	41	15	12 to 74
Urine-specific gravity (no unit)	1.020	1.015–1.020	≥1.025 (*n* = 8)
Urine sodium (mmol/L)	69	53–100	<30 (*n* = 5)
Urine albumin (mg/L)	14	4–52	>100 (*n* = 6)
Urine albumin/creatinine (mg/mmol)	3.4	1.2–9.6	>10 (*n* = 7)
Urine color *	5	3–6	Darkest color 7 (*n* = 6)
**Clinical indices**			
Age (years)	82	9	65 to 97
Body weight (kg)	67	14	44 to 98
Breathing frequency (breaths/min)	16	3	≥20 (*n* = 6)
Thirst severity (VAS scale 0–100)	41	26	<10 (*n* = 5) and > 80 (*n* = 3)
Daily medications (N)	10	7–12	3 to 18
Tongue furrows (graded 1/2)	1.5	-	Present = 1, Absent = 2
Poor skin turgor (graded 1/2)	1.8	-	Present = 1, Absent = 2
**Hemodynamics**			
Heart rate (bpm)	83	13	≥100 (*n* = 6)
Systolic arterial pressure (mmHg)	123	18	≤100 (*n* = 5)
Diastolic arterial pressure (mmHg)	65	11	≤50 (*n* = 11)
Mean arterial pressure (mmHg)	84	12	<70 (*n* = 5)

* Using the scale published by Armstrong et al. [7].
